# Experimental Verification and Evolutionary Origin of 5′-UTR Polyadenylation Sites in *Arabidopsis thaliana*

**DOI:** 10.3389/fpls.2018.00969

**Published:** 2018-07-05

**Authors:** Yingdong Zhu, Jack C. Vaughn

**Affiliations:** Program in Cell Molecular and Structural Biology, Department of Biology, Miami University, Oxford, OH, United States

**Keywords:** 5′-UTR, alternative polyadenylation, exon evolution, exon shuffling, uORF

## Abstract

Messenger RNA (mRNA) polyadenylation is an indispensable step during post-transcriptional pre-mRNA processing for most genes in eukaryotes. The usage of one poly(A) site over another is known as alternative polyadenylation (APA). APA has been implicated in gene expression regulation through its role of selecting the ends of a transcript. Recent studies of polyadenylation profiles in the *Arabidopsis* database unexpectedly predicted that a portion of the poly(A) sites are located in the 5′-UTR, which remains to be experimentally verified. We selected 16 genes from a dataset of 744, based on criteria designed to minimize problems in interpretation. Here, we experimentally verify 5′-UTR-APA in *Arabidopsis* for 10 of the 16 selected genes, and show for the first time existence of independent polyadenylated 5′-UTR transcripts, arising due to alternative polyadenylation. We used 3′-RACE and sequencing to validate poly(A) sites and northern blot to show that the observed short upstream transcripts do not arise from the 3′-end of a previously unrecognized convergent gene. Evidence is reported showing that two of the independent upstream open reading frame (uORF) transcripts studied, one containing a complex dual uORF, very likely arose by exon shuffling following duplication of the 5′-end from the downstream major open reading frame (mORF). Finally, results are presented to show that the uORF in this gene may encode two short functional proteins, based on observation of amino acid sequence conservation encoded by the dual uORFs.

## Introduction

The structure of transcribed regions in a typical eukaryotic protein-encoding gene includes a 5′-UTR, one or more coding exons, and a 3′-UTR. Introns may interrupt any of these regions, and their alternative splicing contributes to the diversity of proteins which may be produced by a gene. The initially transcribed pre-mRNA contains a relatively long 3′-UTR, which is cut at the poly(A)-cleavage site followed by post-transcriptional addition of a poly(A) tail. The poly(A) site is generally located several tens or hundreds of nucleotides downstream of the mRNA stop codon (reviewed in Hunt, 2008). Determination of the cleavage site requires *cis*-acting components, which in some higher animal species include a conserved sequence A-A-U-A-A-A (reviewed in Graber et al., 1999; Zhang et al., 2005). In plants, however, only about 10% of the genes contain this sequence signal, the cleavage site instead depending on the appropriate sequence context around a poly(A) site (Millevoi and Vagner, 2009), in addition to the requirement of a number of *trans*-acting protein factors (Hunt et al., 2012). In eukaryotes, many genes possess more than one poly(A) site, which results in a set of mRNA isoforms which also results in the diversity of proteins generated by a gene. Identification of poly(A) sites serves as a reference point from which to study genes that undergo alternative polyadenylation (APA), a widely-observed mode of gene expression regulation in eukaryotic genomes. For many years, polyadenylation was mainly observed in the 3′-UTR. However, with the emergence of genome-scale approaches in recent years, it has become clear that polyadenylation can occur in other parts of an mRNA in addition to the conventional 3′-UTR (Di Giammartino et al., 2011). If an APA site is located in the 3′-UTR, this will result in transcripts with an unchanged coding potential but the 3′-UTR length will be altered, a circumstance called 3′-UTR-APA. This can influence the fate of an mRNA in several ways, notably by altering its associations with RNA-binding proteins and microRNAs. In contrast, if an APA site is located in the coding region, this will change the mRNA coding potential by producing a different protein isoform, an event which is called CR-APA (Coding Region-APA) (Di Giammartino et al., 2011; Xing and Li, 2011). Thus far, despite some computational methods, lack of poly(A) signal sequence conservation and complexity of the regulatory mechanism still make the exact position of a poly(A) site difficult to predict (Ji et al., 2010; Wu et al., 2015a).

There are a number of *Arabidopsis* datasets with collections of poly(A) sites and analyses of these datasets have elucidated the poly(A) signals at the genome level. The large-scale studies using transcriptome-wide techniques have revealed that more than 70% of *Arabidopsis* genes have multiple poly(A) sites (Wu et al., 2011). About 83% of the poly(A) sites are located in the 3′-UTR, and the remaining 17% in introns, coding regions and 5′-UTRs. Recently, a poly(A) profile dataset of *Arabidopsis thaliana* (wild-type, col) was built (Guo et al., 2016). It included the poly(A) site profiles of leaves, flowers and roots based on the poly(A) tag sequencing (PAT-seq) (Guo et al., 2016) and the direct RNA sequencing (DRS) methods. The poly(A) cluster (PAC), a collection of nearby poly(A) or PAC sites at the whole genome level, were characterized and some preliminary findings were made. The analyses revealed that polyadenylation events can occur in non-3′-UTR regions, including 5′-UTR, introns, coding sequences, antisense and intergenic regions. Interestingly, a portion of the poly(A) sites were predicted to be located in the 5′-UTR. However, their authenticity and function remain to be resolved. The results of that work are reported here.

It has long been held that one of the major distinctions between prokaryotes and eukaryotes, in the processing of genetic information, is that polycistronic mRNAs are widely utilized in prokaryotes, but very rare in eukaryotes (Lewin, 1994). This distinction was shown to be incorrect when many examples of transcribed upstream open reading frames (uORFs) were discovered in different eukaryotic lineages, wherein the bicistronic mature mRNA transcript is composed of one or more short uORFs located in the 5′-UTR in addition to the downstream major open reading frame (mORF). Recently, uORFs were unexpectedly reported in *E. coli* (Beck et al., 2016) and in mammalian mitochondria (Lee et al., 2015; Cobb et al., 2016), which are translated into short peptides, some having regulatory functions. Among eukaryotes, uORFs have been reported in 20–50% of all the mRNA transcripts (Kochetov, 2008), although most of them have not yet been characterized. A comprehensive literature database, uORFdb, has been established to record eukaryotic uORFs (Wethmar et al., 2014).

The uORFs which encode evolutionarily-conserved peptides are relatively rare, comprising <1% of the transcripts in angiosperm plants (Hayden and Jorgensen, 2007; Tran et al., 2008; Jorgensen and Dorantes-Acosta, 2012), *Drosophila* (Hayden and Bosco, 2008), and mammals (Crowe et al., 2006). In the few examples for which a function involving translation of an uORF have been reported, expression regulation of the downstream mORF has been shown to be in response to binding of small effector molecules to the short peptide within the ribosome itself (Lawrence, 2002). The classical example is regulation of the yeast *S. cerevisiae* CPA1 gene by binding of arginine to the uORF-encoded peptide, which stalls the ribosome and prevents translation of the CPA1 coding sequence, perhaps by inhibiting ribosome scanning to the mORF start codon (Lawrence, 2002). Examples in angiosperm plants in which gene expression regulation involving a short uORF-encoded peptide have been reported (Jorgensen and Dorantes-Acosta, 2012), again involving binding of small effector molecules to a short regulatory peptide. In an mRNA transcript containing one or more uORFs, ribosomes may initiate translation at the mORF start codon after leaky scanning through the uORFs, which thereby produce no protein product. Alternatively, an uORF may be translated, followed by reinitiation of the mORF, thus producing two polypeptide products (Wethmar et al., 2014).

The major goal in our research was to study 5′-UTR polyadenylation in *Arabidopsis*, with a primary focus on attempting to verify some of the recently predicted 744 sense poly(A) cleavage sites in the 5′-UTR (Guo et al., 2016). To accomplish this goal, we first selected appropriate poly(A) sites that had been predicted to occur in the 5′-UTR. We then used 3′-RACE to obtain evidence of a poly(A) tail and sequencing to confirm its location in the annotated genes. After demonstrating existence of a poly(A) tail and an upstream tract, we used northern blots to verify that the transcripts did not arise from another gene. We then explored the potential function of the uORFs in the 5′-UTR, based on a BLAST search for evolutionarily conserved amino acid sequences. Finally, we discovered evidences in support of an exon shuffling molecular mechanism for the origins of two of the uORFs. Our long-term objectives are to explore the biological mechanisms underlying 5′-UTR polyadenylation, and the functional significance of what we have termed independent uORFs.

## Materials and methods

### PAT-seq data analysis

The poly(A) tag sequencing (PAT-seq) data was from previous datasets submitted to NCBI, including tender leaf (GenBank no. SRA048565) (Thomas et al., 2012), unopened flower (GenBank no. SRS1295578), and root (GenBank no. SRS1295579) (Guo et al., 2016). All PAT reads from the three PAT-Seq datasets were pooled into one dataset to simplify analysis, and the bioinformatics procedures have been reported elsewhere (Guo et al., 2016). In particular, only poly(A) sites with ≥3 PAT reads were used. Sequences from mitochondria and chloroplasts were ignored, as were sequences from intergenic regions. The surrounding region of a poly(A) site was scanned for ≥8 continuous As to reduce the possibility of internal priming on the genomic sequence. Due to the microheterogeneity of polyadenylation, poly(A) sites located in the vicinity of 24-nt were clustered into one PAC (Ji et al., 2015). For protein-coding genes with multiple transcripts, the longest transcript was used as reference for each gene, and the 3′-end of each transcript was extended for 120-nt in case of missing 3′-UTR poly(A) sites (Wu et al., 2015b). The 5′-UTR PACs were extracted and further characterized based on the features, including location, direction, starting, and ending positions, PAC position, relative expression, distance to the next genes, and gene annotation. In addition, 5′-UTR PACs were separately selected based on their locations in exons or introns in view of the structure of 5′-UTR. In total, 744 predicted sense 5′-UTR PACs had been previously identified in the *Arabidopsis* genome, and were available for our use (Guo et al., 2016). This compilation shows that there are 351 top-strand (+) PACs and 393 bottom-strand (–) PACs. These sites are present within 5′-UTR exons (495 sites) as well as introns (249 sites).

### RNA isolation from plant tissues

*Arabidopsis thaliana* (wild-type, col) was grown in the growth chamber (Percival) at 22–25°C with 16 h light/8 h dark cycle. The tender leaves of 3-week old plants or flowering plants were cut and total RNA was extracted by using an RNA isolation kit (Macherey-Nagel). The quality and quantity of total RNA was checked by NanoDrop spectrophotometer and RNA pico chip (Agilent) prior to use. Poly(A)-enriched mRNA was purified from the total RNA by using oligo-dT cellulose beads (BioLabs), following the directions of the supplier.

### 3′-RACE analysis

To experimentally validate the predicted poly(A) sites in the 5′-UTR, we analyzed the 3′-end of the transcript. Several selected genes with predicted poly(A) sites in the 5′-UTR were studied by 3′-RACE using an RLM-RACE kit (Ambion), as previously described (Ghosh et al., 2013). The cDNA from total RNA was first obtained by reverse-transcription (RT). Gene-specific forward primers were designed by using the primer-BLAST program on the NCBI website (Ye et al., 2012). Outer forward primers were designed for the initial PCRs, followed by a second PCR using nested inner forward primers to increase the signal strength for rare transcripts (Table 1). The transcript of *Arabidopsis* β-tubulin chain 4 (TUB4), which has a 3′-end poly(A) tail, was used as positive control. Adapter and adapter-specific reverse primers were from the kit. The PCR products were analyzed by 2% agarose gel electrophoresis. The resulting bands were excised and DNA purified by using the Wizard SV Gel and PCR clean-up kit (Promega), in comparison with a low molecular weight DNA ladder (BioLabs). The purified products were quantified and then sequenced by Sanger sequencing using a BigDye terminator v3.1 cycle sequencing kit (Applied Biosystems) in the CBFG facility at Miami University. DNA sequences obtained from Sanger sequencing were aligned to the *Arabidopsis* genome to confirm the poly(A) sites.

**Table 1:** Selected genes and gene-specific primers designed for 3′-RACE.

**No**.	**Gene**	**Outer primer**	**Inner primer**
1	AT5G22630	TGGTAGGTGAAGGATTTGACGG	CGGAACTACCCTTCAAGACAC
2	AT4G34110	CGTAATTGTGCTTGTTTCGCC	TTCATCTTCCCTCTCCTCTGAT
3	AT1G71340	TCTCCACCTGCGACATTCTG	CTCTAGTGCCATGTGGGCTT
4	AT4G11830	TGTCTGCGGATGGAAACTTCT	ATGGAAACTTCTAAAGGGTAACATC
5	AT5G20450	AATCCTCCGACTGGTTTCGT	GTTTCTTAGCGGGTGGCTCT
6	AT2G37150	GGCCTAATTGCTTCCTGTTGG	GCACGACGGCTTTATCACTT
7	AT1G13190	CCTCAATTTCACGAGGCAATC	TCGATGTTCTTACTCTCTCGTTG
8	AT4G33300	GGCTTGCTCTTCCTCTCCAA	ACTTCGTAATCTCCAAATTCATCAG
9	AT2G29290	GCCAAAGTCCATGTATCCGAC	AGTGAGTGGTACAATCTGTGATG
10	AT5G06120	CTTATCTCTGCCAGTAGGCTTT	CTCTTGCGCTTCTTTGTAGTATG
11	AT3G13920	AATAGGTTGAGTGGTGTCTATGTT	CTGAGGCAGATATTAATGCTTGTT
12	AT1G03410	GCTAATTAGTGGCGTTTCGAG	CGAGCAACAATGGCGATGAG
13	AT5G61310	GCAACCTCTCCAACAATGAAC	GTGTGTGCAATCTGATGGGT
14	AT2G31150	AGCTGACTTCTGACTACGTTC	GTCTTCCTCTCTCTCTCATAGTCA
15	AT2G16400	TCTCCTTACCACATTTTTACTCTTG	TGACTCCAAGAAACAAGAAGAAGA
16	AT1G54270	TGCTTTCATGGAGAGATAGATGTT	TCAGATATTAATGCTTGTGTTCCTC

### Northern blot analysis

To experimentally validate some of the predicted transcripts, we analyzed the sequences by northern blot using a radioactive method. About 5 ug poly(A)-enriched mRNA was separated in 5% TBE-urea gel under denaturing conditions using an electrophoresis apparatus (BioRad). RNA bands were electroblotted to nylon membranes using an electroblotting apparatus (BioRad). RNA membranes were baked at 50°C for 30 min followed by two cycles of UV crosslink (Spectronics Corp). Gene-specific primers were synthesized (IDT) to amplify the probes complementary to the 5′-UTR of target genes. The probes were labeled with 32-P Ready-To-Go DNA Labeling Beads (Amersham), according to the supplier's directions. RNA membranes were then hybridized with the radioactive probes overnight, followed by wash steps, as previously described (Fetherson et al., 2006). The bands were captured by ImageQuant Tools (Amersham) and results were analyzed, including the band size, alternative products, as well as their relative expression levels. The PCR primer sets for production of northern blot probes were as follows: AT5G20450 (gene no. 5), forward primer, 5′-AATCCTCCGACTGGTTTCGT-3′; reverse primer, 5′-AACGAAAGACG-CTGCCGTG-3′. AT1G13190 (gene no. 7), forward primer, 5′-TCGGATAAGAAAGAGAA-CATCCC-3′; reverse primer, 5′-CTACTTGTTGCTCGACCTTCT-3′. AT2G29290 (gene no. 9), forward primer, 5′-GCCAAAGTCCATGTATCCGAC-3′; reverse primer, 5′-ACTTGCTCAT-TGTCATTTCCATT-3′.

### Function prediction

To identify a potential ORF, we searched for the presence of start and stop codons, and considered the length of the predicted encoded peptides. To discriminate coding and non-coding RNAs, the coding potential was assessed based on sequence features and support vector machine, e.g., Coding Potential Calculator (Kong et al., 2007). To explore possible function, we used Protein-blast in NCBI to do phylogenetic analysis and aligned the protein sequences from different species. Sequence conservation between different species implies a common conserved function, possibly regulatory for this protein. In many cases protein structure is more conserved than protein sequence, and is used to predict functional sites. Likewise, the motifs within protein domains and subcellular localization of the protein can be predicted in SignalP (Petersen et al., 2011).

### Genbank accession numbers and characteristics of verified independent uORF transcripts

GenBank accession numbers have been assigned for the new set of short polyadenylated RNA transcripts described in this study as arising from 5′-UTRs of 10 genes. These are the following:

Gene no. 5, AT5G20450: GenBank no. KY393292; Gene no. 6, AT2G37150: GenBank no. AK227425; Gene no. 7, AT1G13190: GenBank no. KY393293; Gene no. 9, AT2G29290: GenBank no. KY393294; Gene no. 10, AT5G06120; GenBank no. KY393295; Gene no. 11, AT3G13920: GenBank no. KY393296; Gene no. 12, AT1G03410: GenBank no. KY393297; Gene no. 13, AT5G61310: GenBank no. KY393298; Gene no. 14, AT2G31150: GenBank no. KY393299; Gene no. 16, AT1G54270: GenBank no. KY393300. The characteristics of each verified independent uORF polyadenylated transcript are enumerated in Supplementary Dataset II.

## Results

### 5′-UTR PACs selection for experimental confirmation

The PAC sites that uniquely occur in the 5′-UTR of annotated genes were further selected for experimental confirmation. In view of the limitations of computational and large scale analysis, there may be two possibilities: either they are from the 3′-UTR of other genes, or in the 5′-UTR of the host gene. Other than this, some PAC sites are in the sense direction within their annotated genes, some are in the antisense direction, or some may be identified as both sense and antisense when their host genes are overlapping. The antisense polyadenylation has other regulatory functions in gene expression (Pelechano and Steinmetz, 2013), and was eliminated in this study. For these reasons, the preliminary data were arranged by expression level and mapped to the *Arabidopsis* genome browser and reference sequence. The snapshots of PAC positions to genes were obtained by using the genome browser and customer track in The *Arabidopsis* Information Resource (TAIR, www.Arabidopsis.org), and the genome and transcript sequences were from National Center for Biotechnology Information (NCBI, www.ncbi.nlm.nih.gov). Those genes flanked by upstream genes transcribed in the same direction were not considered. Based on the factors, such as length, relative expression level, and distance to other genes, 16 optimal PAC positions that meet the requirements were selected from the potential candidates for experimental confirmation (Table 1).

### Confirmation of 5′-UTR polyadenylation sites by 3′-race

**Figure 1:**
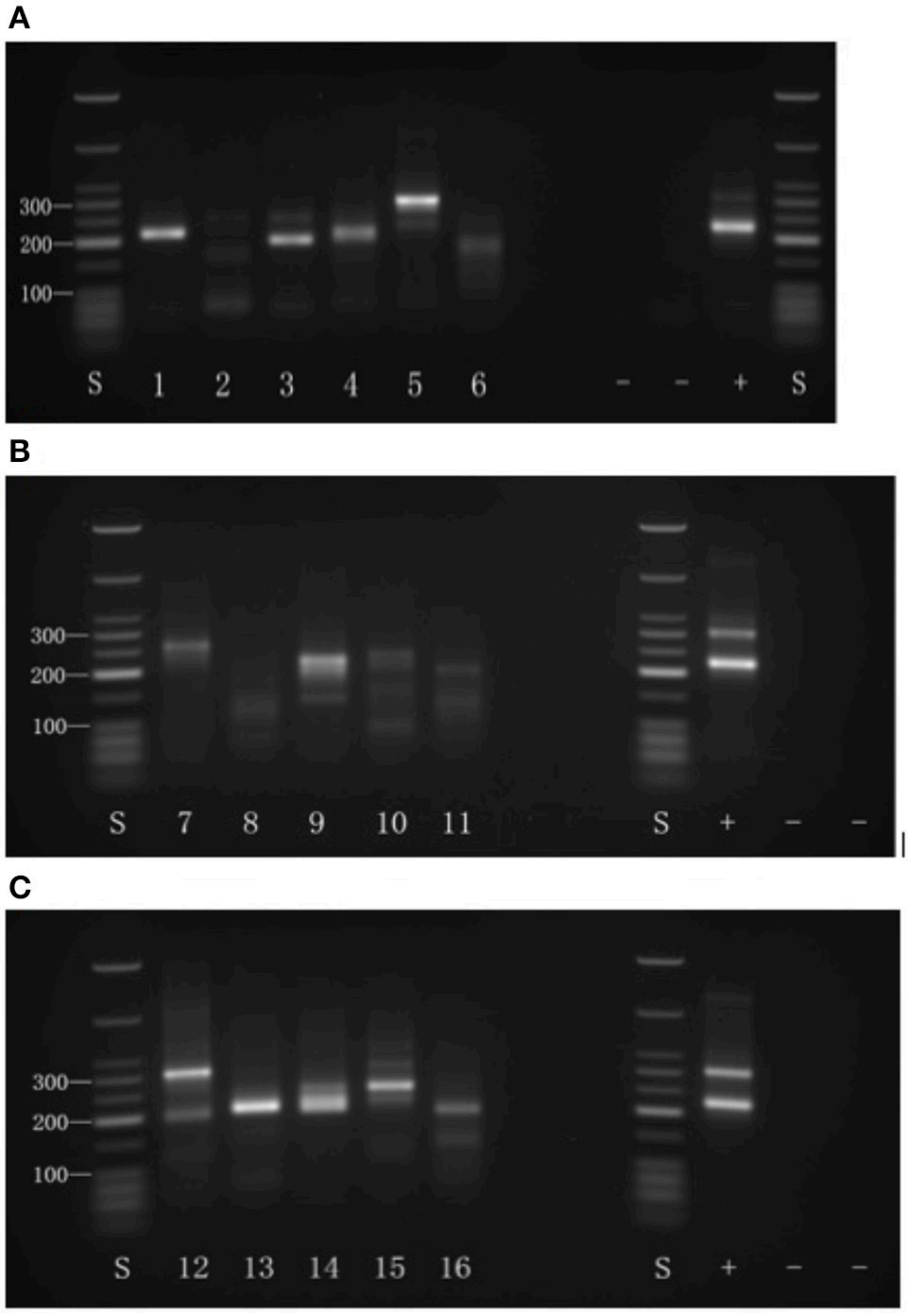
Results of 3′-RACE shown by 2% agarose gel electrophoresis. S, sizer; −: negative control; +: positive control. **(A)** Selected genes #1–6. **(B)** Selected genes #7–11. **(C)** Selected genes #12–16.

**Figure 2:**
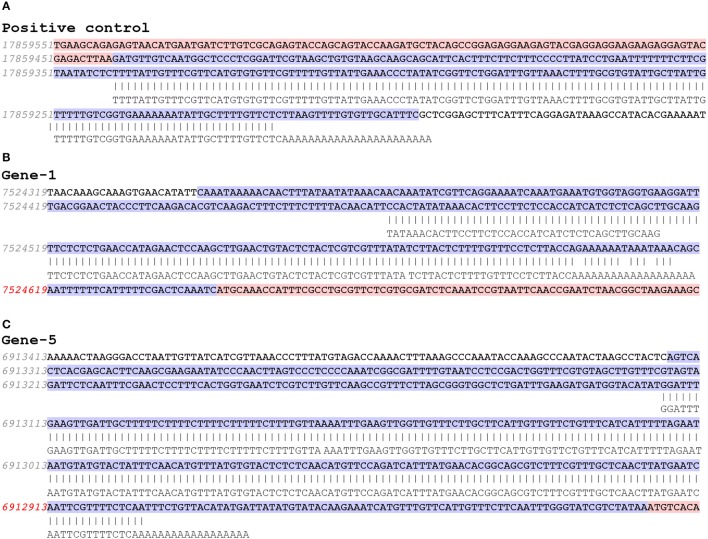
Representative sequence alignment to the *Arabidopsis* genome. DNA sequences are aligned to the *Arabidopsis* genome to confirm the selected poly(A) sites. The upper sequence in each panel is genome sequence, where blue shows 5′-UTR and red shows coding region. Numbers in red show the predicted PAC sites (Guo et al., 2016). The lower aligned sequence in each panel shows sequencing results. **(A)** Positive control is a 3′-UTR poly(A) site for a β-tubulin gene. **(B)** For gene no. 1 (AT5G22630), the poly(A) site is not real in that the band is generated by binding to a long stretch of adenines in the genome sequence. **(C)** For gene no. 5, (AT5G20450) the poly(A) tail is verified, because there isn't a long stretch of adenines in the corresponding genome sequence.

The results of 3′-RACE analysis, following 2% agarose gel electrophoresis, are shown in Figure 1. After excision and sequencing all the 14 bands obtained, most sequencing results agreed with the corresponding poly(A) site in the genome database. Representative results following sequence alignment with the genomic database gene are shown in Figure 2. In the β-tubulin positive control for location of the already known 3′-end poly(A)-tail, the results match the expectation (Figure 2A). A few genes proved to have been poor choices for analysis, as for example gene no. 1 (Figure 2B), where an internal run of A's resulted in a mimic to an authentic 5′-UTR poly(A)-tail. The example shown for gene no. 5 (Figure 2C) gives a good match between predicted PAC and observed poly(A)-tail locations. After genome sequence alignment, 10 PACs out of the 16 were confirmed with a poly(A) tail (Table 2). The complete results for all tested genes that gave interpretable outcomes are shown in Supplementary Dataset I.

**Table 2:** PAC sites in the 5′-UTR confirmed by 3′-RACE followed by sequencing.

**No**.	**Location**	**Start**	**End**	**PAC**	**Expression level**	**Gene**	**Region**
5	Chr5	6912890	6912958	6912913	273	AT5G20450	UTR
6	Chr2	15606647	15606694	15606648	80	AT2G37150	UTR_ intron
7	Chr1	4499266	4499411	4499335	646	AT1G13190	UTR_ intron
9	Chr2	12586290	12586343	12586314	61	AT2G29290	UTR
10	Chr5	1844980	1845038	1845031	49	AT5G06120	UTR_ intron
11	Chr3	4594130	4594133	4594130	48	AT3G13920	UTR_ intron
12	Chr1	846598	846644	846602	36	AT1G03410	UTR
13	Chr5	24654185	24654190	24654185	35	AT5G61310	UTR_ intron
14	Chr2	13272650	13272664	13272651	33	AT2G31150	UTR
16	Chr1	20260492	20260495	20260494	26	AT1G54270	UTR_ intron

*In this compilation, the term “expression” refers to the relative abundance of a particular predicted PAC site as determined in the previously published PAT-seq data analysis (Guo et al., 2016)*.

### Verification of selected 5′-UTR transcripts by northern analysis

**Figure 3:**
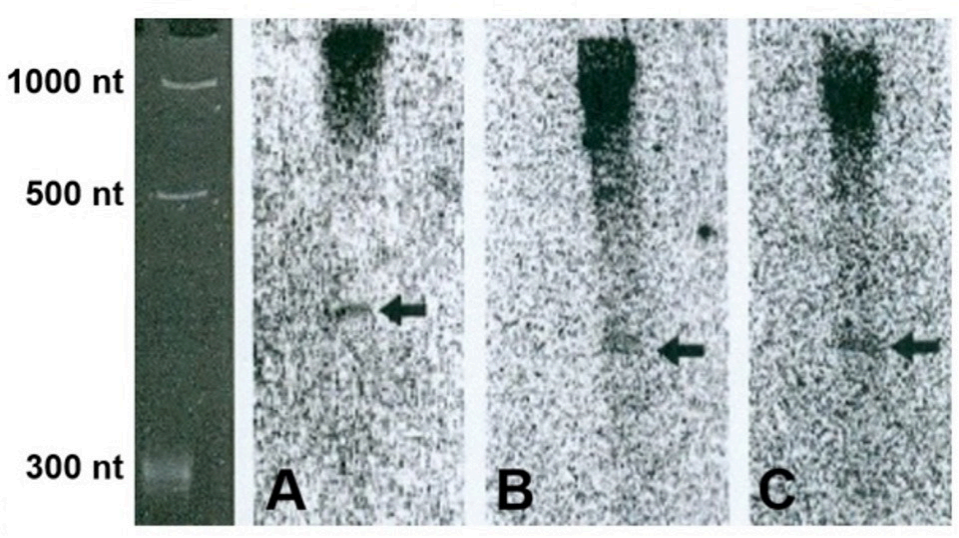
5′-UTR transcripts verified by northern blot. Arrows point to the truncated transcripts. **(A)** Gene no. 5, AT5G20450, **(B)** gene no. 7, AT1G13190, **(C)** gene no. 9, AT2G29290.

It was possible that apparent 5′-UTR poly(A) cleavage sites were due to the 3′-end poly(A) site of a previously unrecognized convergent gene. To eliminate this uncertainty, northern analysis was carried out on PAC sites in 3 different genes, including two in exons and one in an intron, for further confirmation by northern analysis. Only a limited number were analyzed due to limitations in time and resources. Northern blot showed that two bands, in line with the expected sizes, were obtained from two of the three genes tested (Figure 3). The smaller band in Figures 3A,B is interpreted to be the truncated transcript ending at the 5′-UTR poly(A) site, and the longer one to be the full-length bicistronic transcript with probably more than one isoform. However, the smaller band for gene no. 9 in Figure 3C is compatible in size with that expected for transcripts arising from the upstream pseudogene. This is an unresolved uncertainty. As expected, the expression levels of truncated transcript are much lower, in contrast to the high expression level of the full-length transcript, in every case. This result confirms that the 5′-UTR poly(A) sites are not from a previously unrecognized 3′-end of another gene.

### Conserved domains detected in peptides encoded by the confirmed transcripts

**Figure 4:**
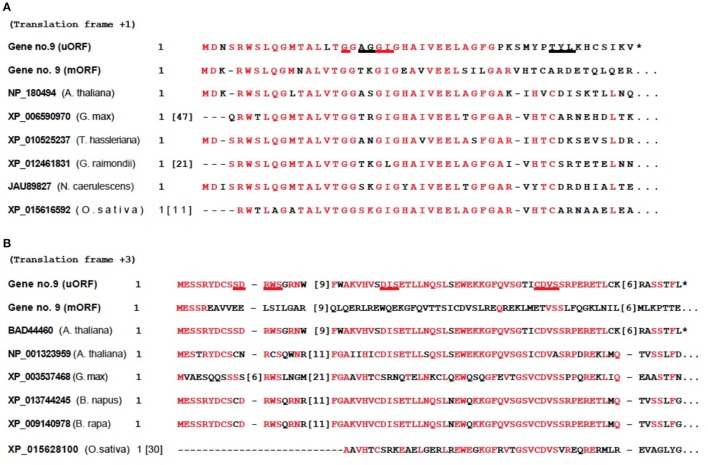
Conserved domains detected in 5′-UTR of AT2G29290 (gene 9). Annotations on the left are NCBI accession numbers and species. Numbers in brackets represent omitted amino acids. Identities between protein sequences are highlighted in red, and potential ligand binding sites are underlined. These proteins are members of the short-chain dehydrogenases/reductases (SDR) family, which belongs to the Rossmann-fold NAD(P)(+)-binding proteins superfamily. **(A)** Nicotinamide-adenine-dinucleotide (NAD), ligand binding sites at G17 A19 G20 G21 I22 T41 Y42 L43. The mORF within the bicistronic 1,833-nt mRNA transcript, GenBank no. NM__001124936 from gene no. 9, was used here. **(B)** Nicotinamide-adenine-dinucleotide phosphate (NADP), ligand binding sites at S10 D11 R12 W13 S14 D36 I37 S38 C61 D62 V63 S64. The mORF within the bicistronic 1,361-nt mRNA transcript, GenBank no. NM_128483 from gene no. 9, was used here. The sequence with accession number BAD44460 from *A. thaliana* is from a polyadenylated uORF short transcript accidentally discovered by others, and is a conceptual translation of the mRNA.

A BLAST search was carried out for each of the transcripts to see if any were conserved between related species. None were found to be conserved, with the exception of gene no. 9. The homology-based method showed two putative conserved peptides encoded by the 5′-UTR transcript from gene no. 9 (Figure 4). Translation analysis showed that these two uORF-encoded peptides use different reading frames. The peptides potentially encoded are members of the SDR family, which belongs to the Rossmann-fold NAD(P)(+)-binding proteins (Hua et al., 2014). The structure/motif-based method showed possible binding sites. The first hypothetical protein has a nicotinamide-adenine-dinucleotide (NAD) ligand (Figure 4A), and the second has a nicotinamide-adenine-dinucleotide phosphate (NADP) ligand (Figure 4B). The evolutionary conservation of the gene no. 9 uORF extends to the rice *O. sativa*, a monocot, with an evolutionary divergence time of about 150 × 10^6^ years (Chaw et al., 2004).

### Evolutionary origin of dual 5′-UTR uORFs in AT2G29290 (gene 9)

**Figure 5:**
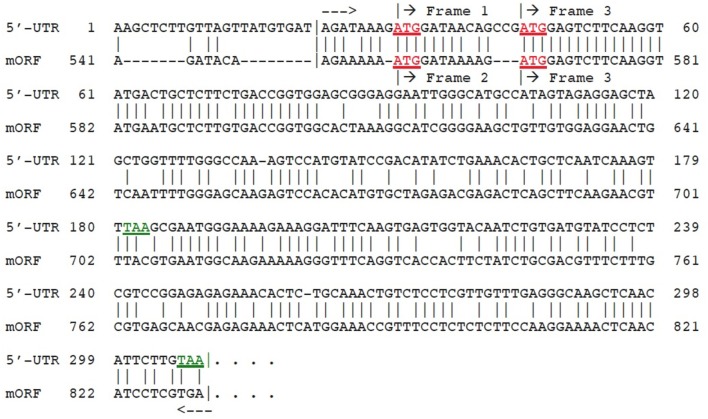
Alignment of 5′-UTR from AT2G29290 (gene 9) with its own mORF. The region of close similarity between the two sequences is bracketed between dashed arrows. Matches are indicated by vertical lines. Start codons (ATG) are indicated in red (underlined), and stop codons (TAA) for the first two ORFs in green. Reading frames as shown by translation analysis are labeled. The region within the brackets in the alignment shows 69% identity between the two sequences. Amino acid sequence predicted to be encoded by 5′-UTR ORF is given in Supplementary Dataset II.

An unusual feature of the dual 5′-UTR uORFs identified in AT2G29290 (gene no. 9), which encodes a member of the NAD(P)-binding Rossmann-fold superfamily of proteins, is that translation analysis shows they utilize two different reading frames. We anticipated that this feature could potentially be useful for identification of the gene from which they arose, perhaps by some sort of duplication event involving a gene located near to AT2G29290. To test this idea, we looked at the structures of genes located adjacent to the subject gene. These genes are: AT2G29270, AT2G29280 (2 short pseudogenes, each comprised of duplicated copies of the 5′-end from the following gene), AT2G29290, AT2G29300, and AT2G29310. One of these genes had a 5′-UTR length exceeding 500-nt, and this was AT2G29290 itself. Surprisingly, translation analysis showed that this gene utilizes two different reading frames in its major ORF (mORF) to produce two alternative mRNA transcripts: GenBank No. NM_128483, 1361-nt using reading frame 2; and NM_001124936, 1833-nt using reading frame 3. This observation suggested the hypothesis that a duplication event involving the 5′-end of the mORF of AT2G29290 may have led to creation of the long 5′-UTR, also containing dual uORFs using two different reading frames. To test this hypothesis, a computer-assisted nucleotide sequence alignment was made between the mORF 5′-end of the gene against its own 5′-UTR. The results (Figure 5) clearly show that the hypothesis is correct. Creation of the necessary new poly(A)-cleavage sites operating in conjunction with the two newly appearing uORFs was likely the result of random sequence change events. Poly(A)-cleavage sites in plants do not contain conserved canonical nucleotide tracts such as the A-A-U-A-A-A sequence that is common in higher animal gene transcripts (reviewed in Graber et al., 1999; Zhang et al., 2005). The necessary new stop codons for the dual uORFs arose by mutations of the original mORF (Figure 5): TAC → TAA (1st uORF) and TGA → TAA (2nd uORF). It has been reported by others that the uORF using reading frame 3 produces an independent polyadenylated transcript (GenBank no. AK176697), in support of our 3′-RACE experimental results. The development of functional poly(A) cleavage sites in plants depends on random mutations and on the nucleotide sequence context in the adjacent pre-mRNA transcript environment (Millevoi and Vagner, 2009).

## Discussion

### Confirmation and characteristics of 5′-UTR polyadenylated uORFs

The major goal in our research was to study 5′-UTR polyadenylation in *Arabidopsis*, with a primary focus on attempting to verify the recently predicted poly(A) cleavage sites for a portion of the 744 predicted sites (Guo et al., 2016). Our studies resulted in identification of a third polyadenylation mode, that of 5′-UTR-APA. In addition, our work resulted in identification of a new category of uORF, which we term the “independent uORF,” distinct from the many previously described uORFs which remain within the body of their partner major ORF gene and which we term the “resident uORF.” Potential consequences of 5′-UTR polyadenylation have been reviewed (Hua et al., 2014). These include RNA degradation, a non-coding RNA expression regulation role, or encoding a short regulatory peptide.

During the course of this study, we were concerned that any short polyadenylated mRNAs detected as apparently arising from the 5′-UTR of a gene could arise from an upstream gene in the same orientation. In selecting the 16 genes for our study from the dataset, we therefore utilized several criteria to eliminate this possibility. The most important was based on information in the genome browser of The Arabidopsis Information Resource, specifically excluding those genes flanked by an upstream gene transcribed in the same direction as the selected gene. Since a gene annotation inevitably may contain errors, we verified several of the short transcripts identified from the 3′-RACE work by northern analysis, using sequence-specific probes. Finally, in addition to the research we have done, these newly discovered 5′-UTR independent polyadenylated uORF transcripts are evidenced by other reports observed by accident in a search of non-annotated *Arabidopsis* cDNAs. This is the case for the mRNA associated with gene AT5G20450 (gene no. 5), the independent 5′-UTR transcript discovered in our study having been assigned GenBank no. DQ108772 by others. In addition, 5′-UTR uORFs which we have found as independent polyadenylated transcripts in our study have been reported by others to be also retained in long bicistronic mRNAs. One example is for gene AT2G37150 (gene no. 6), GenBank no. AK227425. Another example is for gene AT2G29290 (gene no. 9), GenBank no. NC_003071. In the case of this gene, where in our study an independent 5′-UTR polyadenylated transcript was found, a short mRNA has also been reported by others, GenBank no. AK176697, and a hypothetical encoded polypeptide for this sequence has been assigned GenBank no. BAD44460. Overall, this arrangement may be common to every 5′-UTR independent polyadenylated uORF discovered in our study, since the *Arabidoposis* annotated genome sequence tracts are derived from sequencing of EST clones, themselves obtained from cDNAs (Shangguan et al., 2013). These reports of accidental discoveries further support our experimentally derived findings.

### Potential functions for 5′-UTR polyadenylated independent uORFs

The dual uORFs for gene no. 9 arose from a duplication of the 5′-end of the downstream mORF (Figure 5). It is therefore striking that little or no homology in terms of potentially encoded amino acid sequence conservation was detected between the 5′-UTR region of gene no. 9 and the 5′-end of this gene's mORF during the BLAST search for uORF reading frame +3, although the identity was surprisingly higher for frame +1 (Figures 4A,B). With the exception of gene no. 9, none of the genes in our study encode potential uORF peptides that are evolutionarily conserved, and we therefore believe their uORF functions do not include a function for potential encoded peptides. If so, this would place a potential function for these transcripts among the noncoding RNAs in eukaryotic cells (reviewed in Quan et al., 2015), some of which have a 5′-cap and a 3′-polyadenylated tail (Du Toit, 2013). Little is known about their molecular mechanisms of function. In our interpretation, the original protein domain containing NAD and NADP binding sites in gene no. 9 has retained very little of its conserved amino acid sequence. However, following its duplication to produce the dual uORFs, the original amino acid sequence has been highly conserved. This conservation in amino acid sequence in both dual uORFs shows that natural selection has favored their retention, which in turn implies that they have a function, perhaps to produce a short protein important in ligand binding.

The many examples of conventional resident uORFs in diverse eukaryotic lineages described above in the Introduction have obvious similarities to the independent uORFs in our study. We suggest that there is an evolutionary relationship between some 5′-UTR resident uORFs, 5′-UTR independent uORFs and their downstream mORFs, wherein evolution facilitates the formation of independent polyadenylated 5′-UTR uORFs in certain 5′-UTRs, thus promoting the separation of uORF and mORF. Not all 5′-UTRs produce a polyadenylated uORF transcript. This may be explained by the reasons that activation of a poly(A) signal by mutation is random and the polyadenylation machinery is poorly conserved in plants (Shen et al., 2008; Andersen et al., 2012). Such an ORF may mediate transcriptional control, in that it can reduce or eliminate the original transcript in APA. The short transcript would be expected to have a relatively low expression due to the suboptimal location of the polyadenylation elements, as is in fact observed (Guo et al., 2016). Some of these novel 5′-UTR ORFs also exist within a single long mature mRNA, containing both the 5′-UTR ORF sequence and the mORF sequence. In the full-length transcript, the 5′-UTR ORF is qualitatively similar to an uORF in front of the mORF, and hence, it may mediate translational control, by competing with the mORF. Therefore, the potential exists to function as an uORF, and there is also a capacity for the 5′-UTR uORF to function independently as a separate noncoding mRNA. There is, however, another explanation for the origin of some independent uORFs we have studied, which is discussed below.

### Evolutionary origin for independent 5′-UTR polyadenylated uORFs

**Figure 6:**
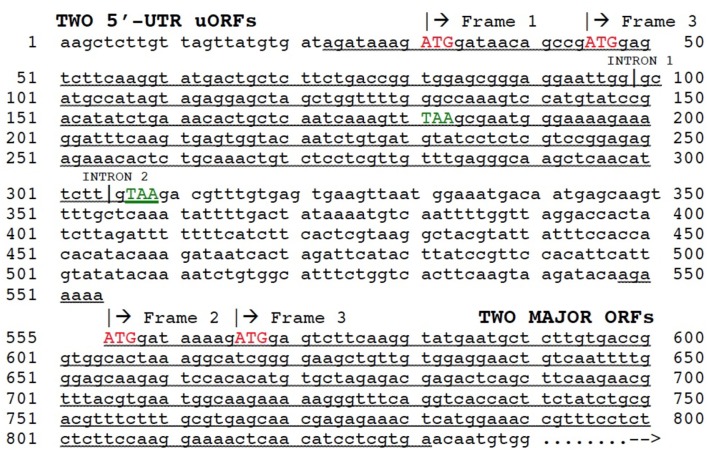
Structure of AT2G29290 (gene 9) mRNA transcript NM_001124936. Regions of duplication (underlined) between 5′-UTR containing two uORFs and 5′-end of major ORF are indicated. Start codons (ATG) are shown in red, and 5′-UTR stop codons (TAA) in green. Positions of the first two introns are marked, although others occur further downstream and are not shown. Reading frames shown by translation analysis are labeled.

**Figure 7:**
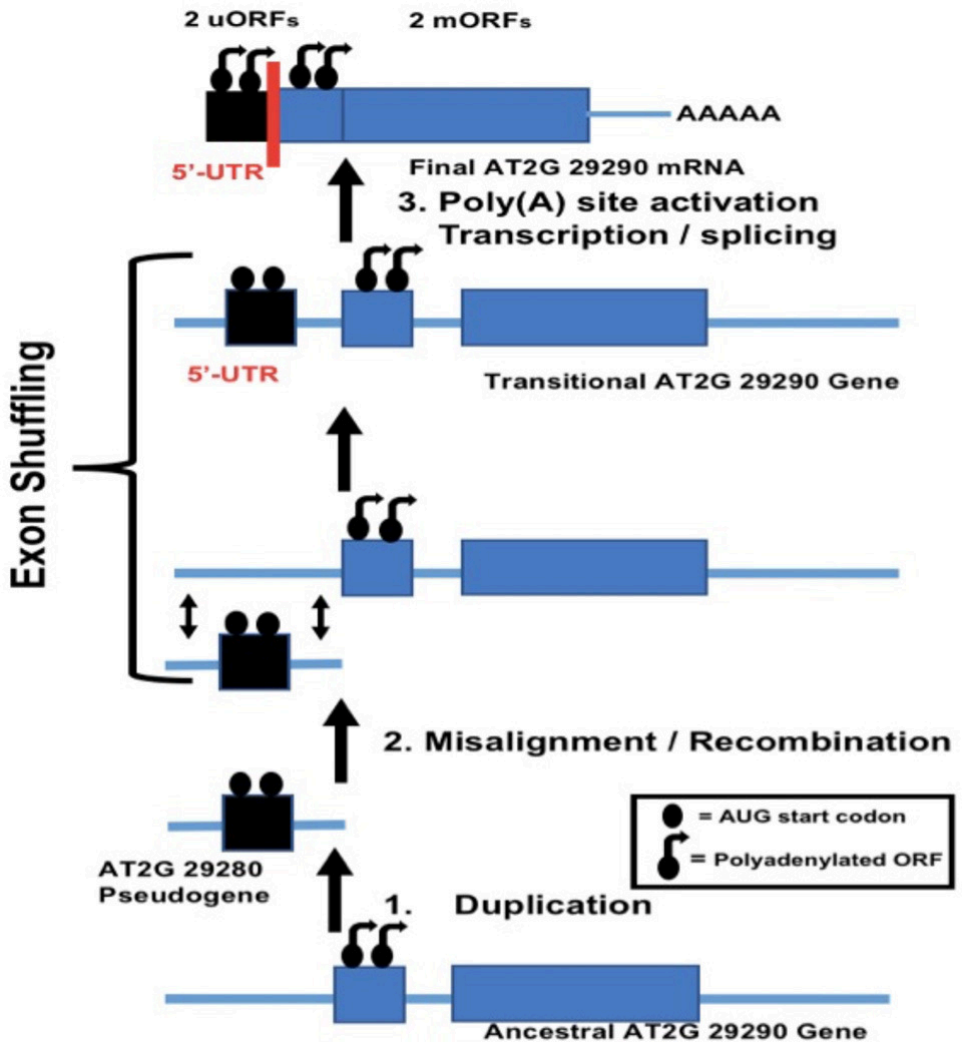
Exon shuffling model for evolutionary origin of dual uORFs in AT2G29290 (gene no. 9). (1) Duplication of a region within the mORF's 5′-end containing the start codon produces a short pseudogene, shown as AT2G29280 in the annotated *Arabidopsis* genome database TAIR. (2) Misalignment and subsequent recombination with the original genomic copy results in a short upstream duplicate of the 5′-end element. (3) Poly(A) site activation and subsequent transcription results in two mRNA products, a short one from the newly-acquired 5′-uORF and the other, a longer mORF, arising from the original gene itself.

The potential role of duplication behind evolutionary origin of the dual uORFs which we have described was based on analysis of mRNA transcript structures, but the mechanism responsible for a new exon combination must have operated at the DNA level. The question arises as to the mechanism for insertion of a duplicated partial 5′-UTR into the gene of its own origin. Exon shuffling is a mechanism for creation of new exon combinations *via* crossing over and recombination, usually within an intron or non-coding region following misalignment between homologs or non-homologs. Exon shuffling can also occur by FB element-mediated transposition (Moschetti et al., 2004). Exon shuffling *via* crossing over and recombination has been repeatedly invoked to explain how plant mitochondrial and chloroplast genes, following their migration into the nucleus, could have acquired a new 5′-end exon containing the targeting signal necessary for subsequent return of a protein product to the mitochondrion or chloroplast (Nugent and Palmer, 1991; Sandoval et al., 2004; Ueda et al., 2007). This mechanism involves crossing over/recombination within an intron flanking the duplicated donor sequence and an intron or non-coding region flanking the host gene. We therefore looked at the structure of gene AT2G29290 in the 5′-UTR region, giving special attention to intron locations. This analysis showed that within the genomic structure an intron is indeed located at the 3′-end margin of the duplicated 5′-UTR nucleotide sequence tract (Figure 6). Surprisingly, as previously mentioned, a short pseudogene, comprised of a duplicated copy of the 5′-end of adjacent gene AT2G29290, was found as well. This likely represents the postulated partial gene duplication in our scenario. Taken together, these results are in complete agreement with an exon shuffling mechanism behind evolutionary origin of the dual 5′-UTR uORFs present in the AT2G29290 gene. A model summarizing the postulated mechanism of origin for the dual uORFs in gene AT2G29290 is shown in Figure 7. We have only presented evidence for evolutionary origin based on the analysis of gene AT2G29290, and do not mean to imply that all gene 5′-UTR uORFs have arisen in this manner.

**Figure 8:**
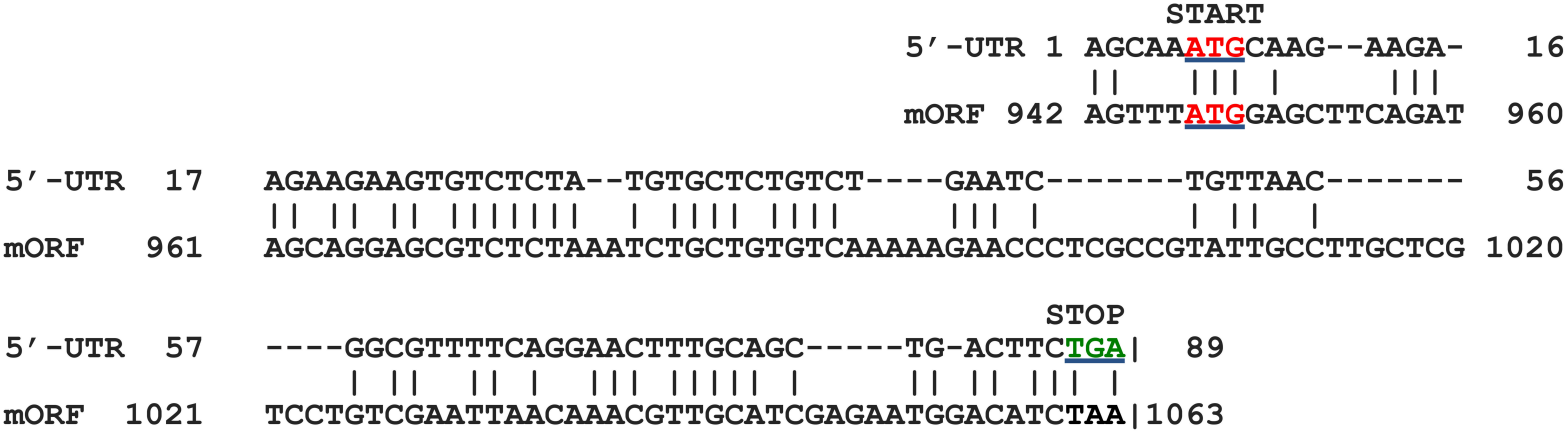
Alignment of 5′-UTR from AT2G31150 (gene 14) with its own mORF. The region of close similarity between the two sequences is shown. Start codons (ATG) are indicated in red (underlined), and stop codons (TGA) for the two ORFs in green. The region shown in the alignment shows 51% identity between the two seqences.shown. Matches are indicated by vertical lines. Amino acid sequence predicted to be encoded by 5′-UTR ORF is given in Supplementary Dataset II.

This exercise was also undertaken for the other genes in our study that were found to contain uORFs. Gene AT2G31150 (gene no. 14) also shows marked similarity between the 5′-UTR sequence and that of the 5′-end from the downstream mORF in the nucleotide alignment (Figure 8). This observation suggests that there may be many examples of independent uORF origin *via* partial gene duplication and exon shuffling in the *Arabidopsis* genome. We do not wish to imply that the origin of some uORFs containing a poly(A)-site in the 5′-UTR is specific to only the independent class of uORFs. It is possible that some conventional 5′-UTR uORFs lacking a poly(A)-site also originated using this pathway, although we have not explored that idea. This is the first report describing a molecular mechanism for how an independent uORF(s) may have originated. It will be interesting to see, in future research, if this mechanism applies to resident uORFs, or to other independent uORFs.

Our study is the first to describe 5′-UTR-APA. The results from this work will be useful for understanding polyadenylation in plants. Firstly, verification of the novel short upstream transcripts will result in improvement of the *Arabidopsis* genome annotation. More importantly, our results may provide new evidence for functions of mRNA polyadenylation resulting in potential gene expression modification in these genes. The next logical step in this study would be to address the biological significance of the 5′-UTR poly(A) sites and ensuing short mRNA transcripts. This could be done by conducting appropriately designed experiments in which mutants are introduced into the selected genes, followed by transfer to the knockout of the endogenous gene background in order to see an effect.

## Data availability statement

Datasets and chromatograms of Sanger sequencing results are available upon request.

## Author contributions

All authors listed have made a substantial, direct and intellectual contribution to the work, and approved it for publication.

### Conflict of interest statement

The authors declare that the research was conducted in the absence of any commercial or financial relationships that could be construed as a potential conflict of interest.
