# Supplementary material for: Experimental Verification and Evolutionary Origin of 5′-UTR Polyadenylation Sites in Arabidopsis thaliana
Source: Front Plant Sci. 2018 Jul 5;9:969. doi: 10.3389/fpls.2018.00969 (PMC6041940; doi:10.3389/fpls.2018.00969)
Supplement: Supplementary file 1 [file Data_Sheet_1.DOCX]

# Submitted to: *Frontiers in Plant Science*

### Experimental verification and evolutionary origin of abundant novel 5’-UTR polyadenylation sites in *Arabidopsis thaliana*

**Supplementary Datasets I – II**

**Yingdong Zhu and Jack C. Vaughn***

***Corresponding author**

**Email: vaughnjc@miamioh.edu**

**Supplementary Dataset I: Polyadenylated 5’-UTR transcripts detected by 3’-RACE**

No.5

Chr5 6912890 6912958  6912913 273  AT5G20450 - five_prime_UTR


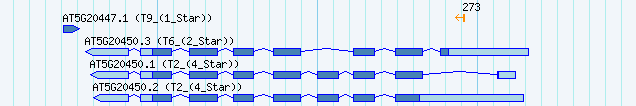


6913313CTCACGAGCACTTCAAGCGAAGAATATCCCAACTTAGTCCCTCCCCAAATCGGCGATTTTGTAATCCTCCGACTGGTTTCGTGTAGCTTGTTTCGTAGTA

6913213GATTCTCAATTTCGAACTCCTTTCACTGGTGAATCTCGTCTTGTTCAAGCCGTTTCTTAGCGGGTGGCTCTGATTTGAAGATGATGGTACATATGGATTT

||||||

GGATTT

6913113GAAGTTGATTGCTTTTTCTTTTCTTTTCTTTTTCTTTTGTTAAAATTTGAAGTTGGTTGTTTCTTGCTTCATTGTTGTTCTGTTTCATCATTTTTAGAAT

||||||||||||||||||||||||||||||||||||||||||||||||||||||||||||||||||||||||||||||||||||||||||||||||||||

GAAGTTGATTGCTTTTTCTTTTCTTTTCTTTTTCTTTTGTTA AAATTTGAAGTTGGTTGTTTCTTGCTTCATTGTTGTTCTGTTTCATCATTTTTAGAAT

6913013AATGTATGTACTATTTCAACATGTTTATGTGTACTCTCTCAACATGTTCCAGATCATTTATGAACACGGCAGCGTCTTTCGTTTGCTCAACTTATGAATC
 ||||||||||||||||||||||||||||||||||||||||||||||||||||||||||||||||||||||||||||||||||||||||||||||||||||

AATGTATGTACTATTTCAACATGTTTATGTGTACTCTCTCAACATGTTCCAGATCATTTATGAACACGGCAGCGTCTTTCGTTTGCTCAACTTATGAATC

6912913AATTCGTTTTCTCAATTTCTGTTACATATGATTATATGTATACAAGAAATCATGTTTGTTCATTGTTTCTTCAATTTGGGTATCGTCTATAAATGTCACA

|||||||||||||||

AATTCGTTTTCTCAAAAAAAAAAAAAAAAAA

No.6

Chr2 15606647 15606694 15606648 80  AT2G37150 - intron_five_prime_UTR


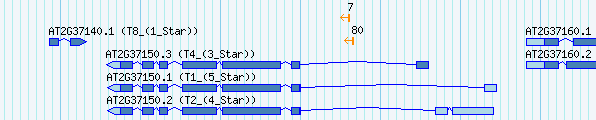


15607048GGAATAGAATTTGGTGTTCTCATTGTATTAGTGAAGTCAAGGGCCTAATTGCTTCCTGTTGGTCTACTTTAAAGGTGTGCAGAAATGATGAATCAGTGAT

15606948CAATCAAGCACGACGGCTTTATCACTTGGGTAGTGTAGTGTATTCGTGGTTAGGACTTTCTAAACCTATATCCTTCTAGGATTATTTGCCTGCAGGTGCA

15606848CTTTTGAGGGTTTCGGTTCAGAAAAATATTTTAAGCTTAAGTCCTGCTATTGGATTTTCCATGTAATTTTCTTGCTCATGTCATCTATGGTTGTAAGTTT

15606748AGTGAAATACATGTACAAGGCCTTTACTTGAATATAAACGAAGAAGTAGTACATTGATAGAAACTGAAAAGATGATATCAAGTGTTACCTATTTTGCATG

||||||||||||||||||||||||||||||||||||||||||||||||||||||||||||||||||||||||||||||||||||||||||||||||||

TTGGGGAAACCTGTCCAGGCCCTTTACTTGAATATAAACGAAGAAGTAGTACATTGATAGAAACTGAAAAGATGATATCAAGTGTTACCTATTTTGCAAA

15606648AACTTGCTCTGATGCTCTTTTATGCGGATACAGTATATGATAATTTGCCATCGTGCCTTATTCTTTCATCCTCTTCTATTTCCTAATCAGTTTCCTATAG

|

AAAAAAAAAAAAAAAAAAAA

No.7

Chr1 4499266 4499411 4499335 646 AT1G13190 + intron_five_prime_UTR


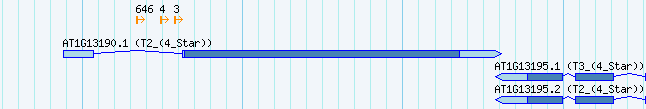


4499035ACGGAGAAGGTCGAGCAACAAGTAGTCAAAGTAAGCAAGCGTAGTTTTTTCTCTCTGTCTTTTTCTTTCCGATCCTCAATTTCACGAGGCAATCAATTCA

4499135ATTCTATTCAATTCGATGTTCTTACTCTCTCGTTGAACTGTAACTTTCCTCGTAGATCTAGTTTGTTTCTTTTCCGATTTTGTAAGAACCCTAATGTTTA

|||||||||||||||||||||||||||||||||||

CTCTCTCGTAAAACTTAGTTTGTTTTCTTTTCCGATTTTGTAAGAACCCTAATGTTTA

4499235TAGATTTCTCGGAAAGGGTTAAAATTTGTGTAGCCCCTTCATTGTAAGTCACACTATGTATGTAATTTGATCTCTTCTATATGTATTTATGAAATCTTGT

||||||||||||||||||||||||||||||||||||||||||||||||||||||||||||||||||||||||||||||||||||||||||||||||||||

TAGATTTCTCGGAAAGGGTTAAAATTTGTGTAGCCCCTTCATTGTAAGTCACACTATGTATGTAATTTGATCTCTTCTATATGTATTTATGAAATCTTGT

4499335AACACCCTTGTTTAAAAAATCGAGCTAGATCCCAAATTTTTCAGATCTGTAATCTAAAGTTCCTCTTTTTTATTTTAATTCGGACATATTGATAATGGCT

||||||||||||||||||||||||||||||||||||

AACACCCTTGTTTAAAAAATCGAGCTAGATCCCAAATTTTAAAAAAAAAAAAAAAAAAAA

No.9

Chr2 12586290 12586343 12586314 61 AT2G29290 + five_prime_UTR


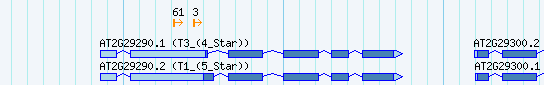


12586014AGGAGTTTATGTTTGTGGAATGTTTTAGGCATGCCATAGTAGAGGAGCTAGCTGGTTTTGGGCCAAAGTCCATGTATCCGACATATCTGAAACACTGCTC

12586114AATCAAAGTTTAAGCGAATGGGAAAAGAAAGGATTTCAAGTGAGTGGTACAATCTGTGATGTATCCTCTCGTCCGGAGAGAGAAACACTCTGCAAACTGT

||||||||||||||||||

GGGGGGGGGAAAAACTCTGGCAAACTGGT

12586214CTCCTCGTTGTTTGAGGGCAAGCTCAACATTCTTGTAAGACGTTTGTGAGTGAAGTTAATGGAAATGACAATGAGCAAGTTTTGCTCAAATATTTTGACT

||||||||||||||||||||||||||||||||||||||||||||||||||||||||||||||||||||||||||||||||||||||||||||||||||||

CTCCTCGTTGTTTGAGGGCAAGCTCAACATTCTTGTAAGACGTTTGTGAGTGAAGTTAATGGAAATGACAATGAGCAAGTTTTGCTCAAATATTTTGACT

12586314ATAAAATGTCAATTTTGGTTAGGACCACTATCTTAGATTTTTTTCATCTTCACTCGTAAGGCTACGTATTATTTCCACCACACATACAAAGATAATCACT

|||||||||||||||||| || ||

ATAAAATGTCAATTTTGGATACGAAAAAAAAAAAAAAAA

No.10

Chr5 1844980 1845038 1845031 49 AT5G06120 + intron_five_prime_UTR


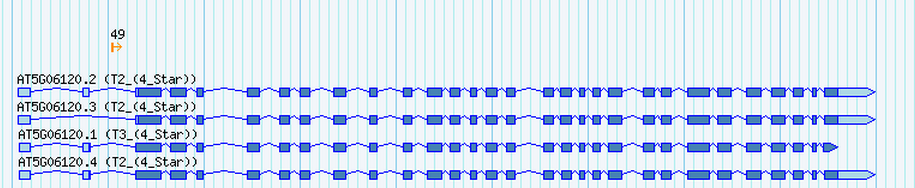


1844731AAACAGAAATCAACCCGAGTACTGAGCTGGAGTAAGGTGATAGACAGCTTGAATACATTTTCCATCATGCAGAGTTAGGAACTTATCTCTGCCAGTAGGC

1844831TTTTACATATTTTTCTAATACTATTTGTGTACCTTCTCTCTCTTGCGCTTCTTTGTAGTATGAGATCTACATGATATTTACCGCTAGCTCACTTGTGTTG

||||||||||||||||||||||||

GAAAATTTTAACGGCTAGCTCACTTGTGTTG

1844931TCTGTGTTAACTCATACTGCACTTTTGTCATTGTTAATGTTGTTCCATCTCTGTTTGGATCTTGGTGTTTCAGTTTTCTTAAGAATGGTTGGTTGCGTTT

||||||||||||||||||||||||||||||||||||||||||||||||||||||||||||||||||||||||||||||||||||||||||||||||||||

TCTGTGTTAACTCATACTGCACTTTTGTCATTGTTAATGTTGTTCCATCTCTGTTTGGATCTTGGTGTTTCAGTTTTCTTAAGAATGGTTGGTTGCGTTT

1845031ACTGTGGAAATGTATCATTTGTTTTTTTTCTCCTGCAACCTGTTTGCCTTATTGTGATATTAAATTTCCAATCCAATTCTAGATATTTTGATTGAAACAG

||||||||||||||||||||||||||||||||||

ACTGTGGAAATGTATCATTTGTAATTGTTCACCTAAAAAAAAAAAAAAAAAAA

No.11

Chr3 4594130 4594133 4594130 48 AT3G13920 - intron_five_prime_UTR


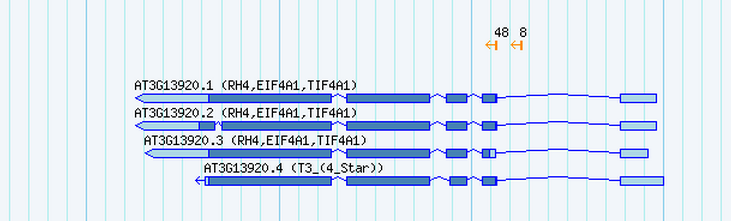


4594430CAGATGTTCTTGTCTTAACACTCACTTGGTTTTGTCTGATTGAATGTTTTTTTTTGTAATTTGGAGAAATAGGTTGAGTGGTGTCTATGTTTAATAGATA

4594330ACCATATGTGCCTTTATACATTTTCTCCGCACATCATCTGAGGCAGATATTAATGCTTGTTTTCCTCTCTTTATGGACTTCGAGTTTGGCTTTCTTGTCA

||||||||||||||||||||||

AAGGGGAATTTCGAGTTTGGCTTTCTTGTCA

4594230CATTTCCTCCTTTGCAATCATATTATTAACGATATCAAAATA-GGTGTTGTCTCTCTTCCTTTTGATATGAAATCTGATTGTTTTTGTTCTTTTTATGTAG

|||||||||||||||||||||||||||||||||||||||||| ||||||||||||||||||||||||||||||||||||||||||||||||||||||||||

CATTTCCTCCTTTGCAATCATATTATTAACGATATCAAAATACGGTGTTGTCTCTCTTCCTTTTGATATGAAATCTGATTGTTTTTGTTCTTTTTATGTAA

4594130CATGGCAGGATCTGCACCAGAAGGCACACAGTTTGATGCACGTCAGTTTGACCAGAAACTCAATGAAGTGTGAGTCTTTTTACTTCTGTTTTTTCATTCT

|

AAAAAAAAAAAAAAAAAA

No.12

Chr1 846598 846644 846602 36 AT1G03410 - five_prime_UTR(2A6)

*
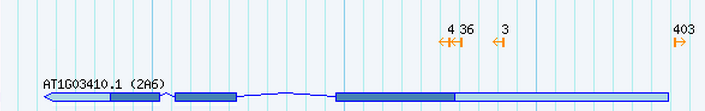
*

846802TTAAATGTTGTTTTGCTAATTAGTGGCGTTTCGAGAGCTAGGCGAGCAACAATGGCGATGAGATTTAATATGGTAAGTGTGTAGTTAATTAACAAAAGTT

|||||||

AAGGGGGGTAGTTAATTTAACAAAAAGTT

846702CAAACCGGATGCTATTCTTACTTGTAAAGACAAACGACCAATTAA-GAATCCAAATTTTAATATAGGAATTGAAATAAACTAATGGACCTTGTTTTTGGCG

||||||||||||||||||||||||||||||||||||||||||||| |||||||||||||||||||||||||||||||||||||||||||||||||||||||

CAAACCGGATGCTATTCTTACTTGTAAAGACAAACGACCAATTAACGAATCCAAATTTTAATATAGGAATTGAAATAAACTAATGGACCTTGTTTTTGGCG

846602ATTCATTTCTTTATTTGTTATCTTAATATGGGTCACGATTCCTTTTGTTATTTAATTGTATTAAGATGTGCATTAAGATGTGGAATAATAGCACTTATGC

|||||

ATACAAAAAAAAAAAAAAAA

No.13

Chr5 24654185 24654190 24654185 35 35 AT5G61310 - intron_five_prime_UTR


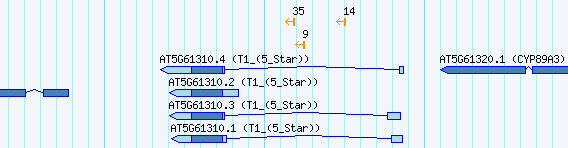


24654485TTGATTTTAATTTAGTTTCGAGGAGACAATTTTTTAACTTAATAGGAGAGGGCCCTTGTCCATGCCTTGAGTCATGGCATCTGTGGCTCCGCAACCTCTC

24654385CAACAATGAACCTGATTCACCACGTGCTTGTGTGTGCAATCTGATGGGTCGATTTTTAAAGGCTTGGGGTTGCTTTCTATTATTGATTGCCAATCTAGAA

|||||||||||||||||||||||||||||||||||

TCCTGGGGGGGGTTGCTTTCTATTATTGATTGCCAATCTAGAA

24654285TTCTCCCTATATACATTGGTCTCCTTGTGCTTCTTTATCATCAATTAGCTGCCCTCATAAGTCCTGCACTGATCAAAGACCTCCTTGACTGATCCACGCG

||||||||||||||||||||||||||||||||||||||||||||||||||||||||||||||||||||||||||||||||||||||||||||||||||||

TTCTCCCTATATACATTGGTCTCCTTGTGCTTCTTTATCATCAATTAGCTGCCCTCATAAGTCCTGCACTGATCAAAGACCTCCTTGACTGATCCACGCA

24654185AAAGTATCTAAATAGTTCTTTGTATTGCTATGTTGAAGTTTGAAGCTTTTTGACAAAGCTTGTTTGATATAGGCATTGCTTGTGGATGATGTTTATGATT

|

AAAAAAAAAAAAAAAAAAAAA

No.14

Chr2 13272650 13272664 13272651 33 AT2G31150 + five_prime_UTR


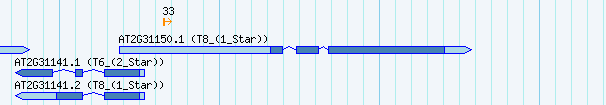


13272351AGTTTTTGAGATAGGAGAGGGGAAAGCAAATGCAAGAAGAAGAAGAAGTGTCTCTATGTGCTCTGTCTGAATCTGTTAACGGCGTTTTCAGGAACTTTGC

13272451AGCTGACTTCTGACTACGTTCCTGATAAATTCTTAGTCTTCCTCTCTCTCTCATAGTCAGAATTGTTTAGTATCTCTGTTTTCGGGCATCGTGTTAGAAT

||||||||||||||||||||||

AATTAATATTTCTGTTTTTTCGGGGCATCGTGTTAGAAT

13272551AATTTGGATAAGCTTTCTATCTGTTTGGAATAGCTTGCCGTTTTGTTATTGTTGTCCACCACTTGTTCGATGAAATGACTGTGTGAAGGTTTTAATTGCT

||||||||||||||||||||||||||||||||||||||||||||||||||||||||||||||||||||||||||||||||||||||||||||||||||||

AATTTGGATAAGCTTTCTATCTGTTTGGAATAGCTTGCCGTTTTGTTATTGTTGTCCACCACTTGTTCGATGAAATGACTGTGTGAAGGTTTTAATTGCT

13272651TTTCGACGGTGTTATTTGCCTCTCTATGGATAACGAAGTTGCATTTCTTGTCAAATTTCAATCCAAAACAAAAATTTGCATTTTTGCTTATGTTAAATTT

||||

TTTCAAAAAAAAAAAAAAAAA

No.16

Chr1 20260492 20260495 20260494 26 AT1G54270 + five_prime_UTR


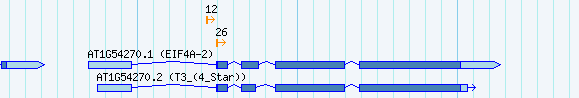


20260194ATCATCCAAAGCTTTTATCTTCGATTTTGGACAACTTTGTGTATATTATATGCTTTCATGGAGAGATAGATGTTTTTGAATGGTATCTGTTATGATAATT

20260294GGATGAACCATATTTCCTATATCTCTCCTGACATCGAATAATTCAGATATTAATGCTTGTGTTCCTCTCTATATGGAACTTCAAGTTTGGCTATTCTGTT

|||||||||||||||

TCAATCCAGGTTTTGGCTATTCTGTT

20260394ACATTCTTACTTTTGCACAACTCTCTCTGGCTATAAGATTTAAGAATCATCTCTAGCTATCTGGGTTCTAAACTATTGTTTCTTTTGTGTATTGTTGTAG

|||||||||||||||||||||||||||||||||||||||||||||||||||||||||||||||||||||||||||||||||||||||||||||||||||

ACATTCTTACTTTTGCACAACTCTCTCTGGCTATAAGATTTAAGAATCATCTCTAGCTATCTGGGTTCTAAACTATTGTTTCTTTTGTGTATTGTTGTAC

20260494TATGGCAGGATCCGCACCGGAAGGAACACAATTTGATACGCGTCAGTTTGACCAGAGGCTGAATGAAGTGTAAGTCCTAAGATCTTTAGTTTCATTACTC

|

AAAAAAAAAAAAAAAAAAAA

**_____________________________________________________________________________________________________________________________________________**

In the upper part of each panel, small hooked arrows represent the predicted poly(A) sites (Guo et al., 2016), and numbers above them depict relative expression levels. The light blue boxes are 5’- or 3’-UTRs, while dark blue boxes are protein coding regions. In the nucleotide sequences, light blue letters represent 5’-UTRs, coding region letters are shown in pink, and introns in green. Sequences shown in black letters are from sequencing after 3’-RACE. Red numbers show predicted locations of poly(A) cleavage sites (Guo et al., 2016).

**Supplementary Dataset II: Characteristics of independent uORF polyadenylated transcripts**

Gene no. 5 (431-nt)

Transcript sequence is nearly identical to GenBank no. DQ108772, and totally inside the 5’-UTR exon of TAIR gene AT5G20450.

1 ctcacgagca cttcaagcga agaatatccc aacttagtcc ctccccaaat cggcgatttt gtaatcctcc

71 gactggtttc gtgtagcttg tttcgtagta gattctcaat ttcgaactcc tttcactggt gaatctcgtc

141 ttgttcaagc cgtttcttag cgggtggctc tgatttgaag atgatggtac atatggattt gaagttgatt

211 gctttttctt ttcttttctt tttcttttgt taaaatttga agttggttgt ttcttgcttc attgttgttc

281 tgtttcatca tttttagaat aatgtatgta ctatttcaac atgtttatgt gtactctctc aacatgttcc

351 agatcattta tgaacacggc agcgtctttc gtttgctcaa cttatgaatc aattcgtttt ctcaaaaaaa

421 aaaaaaaaaa a

Hypothetical protein, translation: CDS: 302-397

MYVLFQHVYVYSLNMFQIIYEHGSVFRLLNL*

Gene no. 6 (1,778-nt)

Transcript sequence is identical to GenBank no. AK227425, which that entry says encodes a “hypothetical protein.” The nucleotide tract is within an intron found in the 5’-UTR of TAIR gene AT2G37150, and is thus a nested 5’-UTR intronic ORF.

Gene no. 7 (>299-nt)

Transcript sequence is within an intron found in the 5’-UTR of TAIR gene AT1G13190, and is thus a nested 5’-UTR intronic ORF. The 5’-end of the transcript is unknown.

1 ...cctcaat ttcacgaggc aatcaattca attctattca attcgatgtt cttactctct cgttgaactg

68 taactttcct cgtagatcta gtttgtttct tttccgattt tgtaagaacc ctaatgttta tagatttctc

138 ggaaagggtt aaaatttgtg tagccccttc attgtaagtc acactatgta tgtaatttga tctcttctat

208 atgtatttat gaaatcttgt aacacccttg tttaaaaaat cgagctagat cccaaatttt aaaaaaaaaa

278 aaaaaaaaaa

Gene no. 9 (409-nt)

Transcript sequence is totally inside the 5’-UTR exon of TAIR gene AT2G29290. Transcript is nearly identical to GenBank no. AK176697. Dual start codons in different reading frames enable the transcript to encode two different evolutionarily conserved peptides.

1 aagctcttgt tagttatgtg atagataaag atggataaca gccgatggag tcttcaaggt atgactgctc

71 ttctgaccgg tggagcggga ggaattgggc atgccatagt agaggagcta gctggttttg ggccaaagtc

141 catgtatccg acatatctga aacactgctc aatcaaagtt taagcgaatg ggaaaagaaa ggatttcaag

211 tgagtggtac aatctgtgat gtatcctctc gtccggagag agaaacactc tgcaaactgt ctcctcgttg

281 tttgagggca agctcaacat tcttgtaaga cgtttgtgag tgaagttaat ggaaatgaca atgagcaagt

351 tttgctcaaa tattttgact ataaaatgtc aattttggtt aggaaaaaaa aaaaaaaaa

1^st^ translation (50-amino acids): 1^st^ CDS: 31-183

MDNSRWSLQGMTALLTGGAGGIGHAIVEELAGFGPKSMYPTYLKHCSIKV*

2^nd^ translation (87-amino acids): 2^nd^ CDS: 45-308

MESSRYDCSSDRWSGRNWACHSRGASWFWAKVHVSDISETLLNQSLSEWEKKGFQVSGTICDVSSRPERETLCKLSPRCLRASSTFL*

Gene no. 10 (>727-nt)

Transcript sequence is within an intron found in the 5’-UTR of TAIR gene AT5G06120, and is thus a nested 5’-UTR intronic ORF. The 5’-end of the transcript is unknown.

1 ...ttttcta tgtgttcatt tctccttact gtttccgttt tctgtgctca agtggttatg attatccttg

68 cacagtatcg aatggttacc actgctattg tctacatact tcgtttattg tttcatcaac acatttttca

138 ttgcgctttc ataaaatcag ataggattta gctgtggcgg aatgatactg taaagtctga gcttaatatc

208 gtctaagagt tttgtttctc ttccatttct gctgtaatta gcttatatag cagcattttg ataatgtgtg

278 gtctcactta gagttgtgtg tcagtccggc gttttgtatg actagtatga gtatttacat gggtggtttg

348 gcaactgaat tttgattttt ggcctttaaa cagaaatcaa cccgagtact gagctggagt aaggtgatag

418 acagcttgaa tacattttcc atcatgcaga gttaggaact tatctctgcc agtaggcttt tacatatttt

488 tctaatacta tttgtgtacc ttctctctct tgcgcttctt tgtagtatga gatctacatg atatttaccg

558 ctagctcact tgtgttgtct gtgttaactc atactgcact tttgtcattg ttaatgttgt tccatctctg

628 tttggatctt ggtgtttcag ttttcttaag aatggttggt tgcgtttact gtggaaatgt atcatttgta

698 attgttcacc taaaaaaaaa aaaaaaaaaa

Hypothetical protein, translation: CDS: 7-168

MCSFLLTVSVFCAQVVMIILAQYRMVTTAIVYILRLLFHQHIFHCAFIKSDRI*

Gene no. 11 (>260-nt)

Transcript sequence is within an intron found in the 5’-UTR of TAIR gene AT3G13920, and is thus a nested 5’-UTR intronic ORF. The 5’-end of the transcript is unknown.

1 ...aataggt tgagtggtgt ctatgtttaa tagataacca tatgtgcctt tatacatttt ctccgcacat

68 catctgaggc agatattaat gcttgttttc ctctctttat ggacttcgag tttggctttc ttgtcacatt

138 tcctcctttg caatcatatt attaacgata tcaaaatagg tgttgtctct cttccttttg atatgaaatc

208 tgattgtttt tgttcttttt atgtaaaaaa aaaaaaaaaa aaaa

Gene no. 12 (1,102-nt)

Transcript sequence is totally inside the 5’-UTR exon of TAIR gene AT1G03410.

1. aaaaaaaaag gtccagtccc aattgggctt aggcccggaa ttacaatcgt ccacagttac aaaacaggaa
2. aaatccggtc accggaaaaa gagaaccaga ggtggatttg ggcagtggaa cggaggcagc aaatagatct

141 attagtaccg tcgtatacaa ggcccactac tttgcaacac aaccaccgga acgagatcat tggccgaagt 211 atacgagtcc tgccgcaaat ctcttaccaa tgagctggcc ggacctgcaa caaacctaac ggaaaaaacc 281 cagtttccga cactccgcca cccaatttgg tcgagtcccc aacctcgcaa aacgaccacg catttccaag 351 cctctgacct ccaaaacctt agatctgaaa attttccaaa agcaaagcaa ccttcaatcc ggattgatgg 421 agacaaaaaa cggaggctaa agaagggtga tggctcagac aacccttcag caaccggaac ccgcaacagc 491 catctcagat ccagtttgtt aaccatcaaa cacgcactga aaacccacca atccacagcg tcttccgcca 561 cagctacagc gtctaaccaa gtggttgaac caagaaagac ttcaccgatc tccaccgacg tccataacca 631 ggaagaaacg gaagagaagc ctctcatcca ccgagaaagg gccaccgctg ccacgcttac cggaaaaacc 701 acaccactcc aatgccggag aagatagctt gccttactcc tgctttgtct tgcaaattca taacctaaga 771 tgaagaacag aggcagaaaa aactagatct gtaaaaactg gaaaaaactt caaagagaga gaaaatgaac 841 agtagcggaa cgattgtcca aagtcctcca aacgagatat ctttaaatgt tgttttgcta attagtggcg 911 tttcgagagc taggcgagca acaatggcga tgagatttaa tatggtaagt gtgtagttaa ttaacaaaag 981 ttcaaaccgg atgctattct tacttgtaaa gacaaacgac caattaagaa tccaaatttt aatataggaa 1051 ttgaaataaa ctaatggacc ttgtttttgg cgattcaaaa aaaaaaaaaa aa

Hypothetical protein, translation: CDS: 835-966

MNSSGTIVQSPPNEISLNVVLLISGVSRARRATMAMRFNMVSV*

Gene no. 13 (>231-nt)

Transcript sequence is within an intron found in the 5’-UTR of TAIR gene AT5G61310, and is thus a nested 5’-UTR intronic ORF. The 5’-end of the transcript is unknown.

1 ...gcaacct ctccaacaat gaacctgatt caccacgtgc ttgtgtgtgc aatctgatgg gtcgattttt

68 aaaggcttgg ggttgctttc tattattgat tgccaatcta gaattctccc tatatacatt ggtctccttg

138 tgcttcttta tcatcaatta gctgccctca taagtcctgc actgatcaaa gacctccttg actgatccac

208 gcaaaaaaaa aaaaaaaaaa aaaa

Hypothetical protein, translation: CDS: 57-158

MGRFLKAWGCFLLLIANLEFSLYTLVSLCFFIIN*

Gene no. 14 (297-nt)

Transcript sequence is totally inside the 5’-UTR exon of TAIR gene AT2G31150.

1 agcaaatgca agaagaagaa gaagtgtctc tatgtgctct gtctgaatct gttaacggcg ttttcaggaa

71 ctttgcagct gacttctgac tacgttcctg ataaattctt agtcttcctc tctctctcat agtcagaatt

141 gtttagtatc tctgttttcg ggcatcgtgt tagaataatt tggataagct ttctatctgt ttggaatagc

211 ttgccgtttt gttattgttg tccaccactt gttcgatgaa atgactgtgt gaaggtttta attgcttttc

281 aaaaaaaaaa aaaaaaa

Hypothetical protein, translation: CDS: 6-89

MQEEEEVSLCALSESVNGVFRNFAADF*

Gene no. 16 (>280-nt)

Transcript sequence is within an intron found in the 5’-UTR of TAIR gene AT1G54270, and is thus a nested 5’-UTR intronic ORF. The 5’-end of the transcript is unknown.

1 ...tgctttc atggagagat agatgttttt gaatggtatc tgttatgata attggatgaa ccatatttcc

68 tatatctctc ctgacatcga ataattcaga tattaatgct tgtgttcctc tctatatgga acttcaagtt

138 tggctattct gttacattct tacttttgca caactctctc tggctataag atttaagaat catctctagc

208 tatctgggtt ctaaactatt gtttcttttg tgtattgttg tagtaaaaaa aaaaaaaaaa aaaa
